# Mechanisms of Neurorespiratory Toxicity Induced by Fentanyl Analogs—Lessons from Animal Studies

**DOI:** 10.3390/ph16030382

**Published:** 2023-03-02

**Authors:** Karam Chamoun, Lucie Chevillard, Aline Hajj, Jacques Callebert, Bruno Mégarbane

**Affiliations:** 1 Inserm, UMR-S1144, Paris Cité University, 75006 Paris, France; 2 Faculty of Pharmacy, Saint-Joseph University, Beirut 1100, Lebanon; 3 Laboratory of Pharmacology, Clinical Pharmacy, and Medicine Quality Control, Saint-Joseph University, Beirut 1100, Lebanon; 4 Research Center, Quebec University Hospital, Laval University, Québec, QC G1V 0A6, Canada; 5 Laboratory of Biochemistry and Molecular Biology, AP-HP, Lariboisière Hospital, 75010 Paris, France; 6 Department of Medical and Toxicological Critical Care, Lariboisière Hospital, Federation of Toxicology APHP, 75010 Paris, France

**Keywords:** fentanyl, naloxone, neurorespiratory effect, opioid, poisoning, toxicity

## Abstract

In 2020, fentanyl and its analogs contributed to ~65% of drug-attributed fatalities in the USA, with a threatening increasing trend during the last ten years. These synthetic opioids used as potent analgesics in human and veterinary medicine have been diverted to recreational aims, illegally produced and sold. Like all opioids, central nervous system depression resulting from overdose or misuse of fentanyl analogs is characterized clinically by the onset of consciousness impairment, pinpoint miosis and bradypnea. However, contrasting with what observed with most opioids, thoracic rigidity may occur rapidly with fentanyl analogs, contributing to increasing the risk of death in the absence of immediate life support. Various mechanisms have been proposed to explain this particularity associated with fentanyl analogs, including the activation of noradrenergic and glutamatergic coerulospinal neurons and dopaminergic basal ganglia neurons. Due to the high affinities to the mu-opioid receptor, the need for more elevated naloxone doses than usually required in morphine overdose to reverse the neurorespiratory depression induced by fentanyl analogs has been questioned. This review on the neurorespiratory toxicity of fentanyl and analogs highlights the need for specific research focused on these agents to better understand the involved mechanisms of toxicity and develop dedicated strategies to limit the resulting fatalities.

## 1. Introduction

According to the American Center for Disease Control and the National Institute of Drug Abuse, opioids represent the major source of drug-attributed fatalities in the USA, with an increasing contribution of fentanyl and fentanyl analogs accounting for more than 65% of the 92,000 fatalities in 1999–2020 [1,2]. A major opioid overdose crisis has been developing in the USA and most occidental countries for 20 years in relation to: (i) an excessive prescription of opioid analgesics without adequate monitoring; (ii) the subsequent development of dependence among opioid-treated patients, leading to abuse or misuse (e.g., crushed-tablet injection and manipulated transdermal patch sniffing); and (iii) more recently, the growing availability of fentanyl, fentanyl analogs and unrelated compounds classified as novel psychoactive substances (NPS), sold on the internet as recreational drugs or pharmaceutical candidates [3]. 

**Figure 1:**
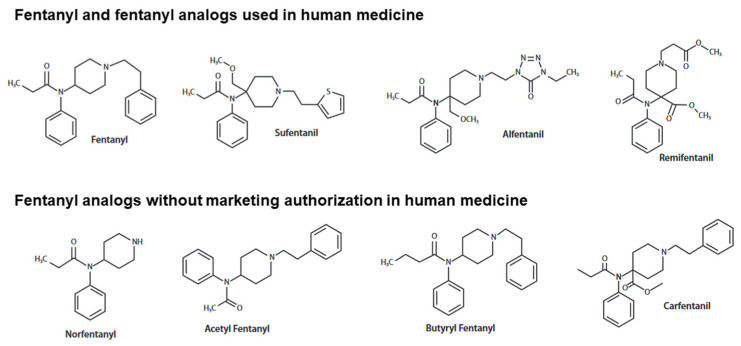
Chemical structure of fentanyl and the main fentanyl analogs.

Fentanyl is a potent synthetic opioid receptor agonist developed in the 1960s. Due to its potency (~50–100 times more potent analgesic activity than morphine), rapid action and short elimination half-life, fentanyl has been used as a step-III pain reliever especially in cancer treatment and palliative and intensive care. More potent synthetic fentanyl analogs such as sufentanil, alfentanil and carfentanil have been also marketed as analgesics or anesthetics for human and/or veterinary medicine. Recently, an extensive number of analogs identified (Figure 1) as designer drugs with or without fentanyl-related chemical structures, such as U-47700, has spread on the recreational scene to bypass toxicological screening tests and laws banning illicit psychoactive substances.

These opioids exhibit similar or enhanced potencies in comparison to the parent fentanyl but have been associated with increased medical concerns [4]. To identify these derivatives and their metabolites in biological fluids and matrices, sophisticated analytical methods for screening and confirmation were developed. However, to date, only 24% of fentanyl analogs can be detected by the most commonly used gas/liquid chromatography-coupled-to-mass-spectrometry assays, most likely due to the low sensitivity of the assays and/or to the poor volatility of the compounds precluding the use of gas chromatography [5]. The development of ultra/high-performance liquid chromatography coupled to tandem mass spectrometry (UHPLC-MS/MS) assays has thus been encouraged. Among those fentanyl analogs, the World Health Organization (WHO) has requested to focus attention on carfentanil, acetylfentanyl, acryloylfentanyl, butyrylfentanyl, furanylfentanyl and ocfentanil. These molecules are potent synthetic opioids to such an extent that only limited amounts are required to cause severe toxicity. Like all opioids, their adverse effects include vomiting/nausea, dizziness, potential abuse and dependence liability and overdose or misuse resulting in consciousness loss and respiratory depression [6]. 

An overdose of fentanyl and analogs results in the classical opioid toxidrome, combining consciousness impairment, pinpoint miosis and bradypnea. Dose-dependent respiratory depression is the most alarming adverse effect resulting from central nervous system (CNS) depression [7]. The onset of thoracic and diaphragmatic rigidity named “wooden chest syndrome” in addition to the classical opioid-related CNS depression has been suggested to explain the enhanced toxicity attributed to fentanyl and its analogs. As a result, an increased risk of death in the absence of immediate management and the requirement of larger doses of naloxone, the non-specific mu-, kappa- and delta-opioid receptor antagonist used as an antidote to reverse opioid-related toxicity in humans, have been questioned, although remaining still controversial [8,9]. Here, we aimed to review experimental studies focusing on the mechanisms of neurorespiratory toxicity attributed to fentanyl and analogs, its reversal using naloxone and the possibility of alternative therapeutic targets for poisoned patient management in the future. 

## 2. Methods

Our research was limited to preclinical studies investigating the neurorespiratory effects produced by fentanyl and its analogs. Databases including PubMed, Google Scholar, Cochrane Library, ISI Web of Science, Embase and Scopus were systematically used for searching scientific articles written in English and French languages and published from 1980/01/01 until 2023/01/31, by using combinations of the following keywords: “Fentanyl” OR “Carfentanil” OR “Sufentanil” OR “Alfentanil” OR “Opiates” OR “Naloxone” OR “Morphine” AND (“Intoxication” OR “Overdose” OR “Neurorespiratory” OR “Treatment” OR “Analogs”. The reference lists of the retrieved publications were thoroughly checked to complete the search. After the exclusion of duplicates, related articles were identified based on title and abstract examination. Then, full-text articles were downloaded and evaluated by two investigators, independently. The references cited in the retrieved articles as well as review articles published in the field were checked to find additional relevant studies. We included in the review all articles reporting preclinical experimental data on the neurorespiratory effects of fentanyl and its analogs. We excluded all articles reporting human data or experimental data focused on non-neurorespiratory effects. 

In this narrative review, we first focused on the neurorespiratory effects resulting from fentanyl and analogs, based on the available experimental data, mainly obtained with fentanyl. Then, we considered the specificities attributed to the main fentanyl analogs if reported. Finally, we discussed the expected response of fentanyl-induced neurorespiratory effects to naloxone and the possible benefits of alternative strategies to reverse CNS toxicity.

## 3. Neurorespiratory Effects of Fentanyl and Analogs

### 3.1. Depression of the Ventilation Command

The fentanyl-related neurorespiratory effects are dose-dependent. Mice exposed to low-dose fentanyl in aerosol (2.7 µg/m^3^) rapidly recovered with no fatality, while 100% of the mice died at higher doses (23.6 µg/m^3^) [10]. No sensory/pulmonary irritation or airway restriction was present, and CNS ventilatory depression was assumed as the cause of death. In the rat, intraperitoneal (IP) fentanyl administered at 80% of its lethal dose, 50% (LD_50_) significantly decreased PaO_2_, increased PaCO_2_, decreased blood bicarbonate and increased blood lactate [11]. Fentanyl (0.05–1.35 mg/kg) administered IP depressed the respiratory rate (RR), the tidal volume (V_T_) and subsequently the minute volume (V_E_) in a dose-dependent manner [12]. All tested opioids administered at 80% of their LD_50_ significantly increased the inspiratory time (T_I_), but fentanyl, similarly to methadone, additionally increased the expiratory time (T_E_) [11]. Consistent with these observations, another study using a smaller intravenous (IV) fentanyl dose (25 µg/kg) elicited a rise in T_I_ and a reduction in V_E_ by decreasing RR, V_T_, the end-inspiratory pause (EIP), the peak inspiratory (PIF) and the peak expiratory flows (PEF) [12]. However, T_E_ was unaffected at that lower dose. The decreases in V_T_ and PEF were relatively transient, whereas the decreases in RR and consequently in V_E_ were more sustained. The increases in T_I_, EIP and PIF lasted for at least 20 min. These depressant effects are mediated by the μ-opioid receptor [13].

Central mu-opioid receptors, particularly those located in the pre-Bötzinger complex, are fundamental to integrally exhibit the onset of the fentanyl-induced switch of pulmonary rapid shallow breathing mediated by C-fibers into apnea [14,15]. The peripheral μ-opioid receptors also contribute to this switch. Fentanyl-associated deficits in respiratory patterning result from a reduced activity of pontine inspiratory neurons, while apnea is observed after the loss of all phasic pontine activity and sustained tonic expiratory neuron activity. Using in situ rat preparations of the arterially perfused dorsolateral pons, neurons were categorized based on their respiratory-associated discharge pattern after incubation with an apneic fentanyl concentration [16]. When exposed to fentanyl, the inspiratory neurons were silenced or exhibited a reduced firing frequency, while the expiratory neurons only reduced their tonic firing frequency. The alterations were reversed when adding naloxone to the preparation. Pontine late-inspiratory and post-inspiratory neuronal activity were absent from apneustic-like breaths during the transition to fentanyl-induced apnea and naloxone-mediated transition to recovery. Interestingly, in vivo in the rat, the profound and immediate inhibition of breathing produced by the highest fentanyl doses was counteracted by the rapid emergence of an opioid-insensitive slow breathing rhythm maintained for a minimum of 30 min, despite coma remaining in the rats [17]. These findings support the existence of respiratory neurons insensitive to opioids able to escape quickly from the respiratory depression caused by fentanyl by maintaining a minimum of respiratory activity. 

When considering fentanyl-attributed neurorespiratory effects, the pharmacokinetic issue of blood–brain barrier (BBB) penetration should also be considered. In the rat receiving a high IV fentanyl dose (80 µg/kg), inhibition of P-glycoprotein (P-gp), a membrane transporter contributing to the efflux of the drug, markedly enhanced the resulting respiratory toxicity, which even led to animal death [18]. These observations strongly support the crucial contribution of P-gp to fentanyl penetration across the BBB and to the amplitude of its subsequent toxicity, suggesting the necessity of close patient monitoring when fentanyl is prescribed alongside with P-gp inhibitors.

### 3.2. Chest Wall Rigidity

In humans, chest wall rigidity has been reported as a rare complication resulting from fentanyl or analog administration in the perioperative or critical care setting, decreasing chest wall compliance and resulting in unsuccessful spontaneous ventilation that challenges the withdrawal from mechanical ventilation [19]. Mainly reported with fentanyl, the occurrence of chest wall rigidity appears dependent on the administered dose and the infusion rate [9]. The closure of glottis and supraglottic structures also contributes to respiratory failure resulting from chest wall rigidity, as reported in anesthesia using synthetic opioids [20]. The studies focused on fentanyl analogs are still limited, but caution, at least in the perioperative field, has been recommended in patients at risk of opioid use disorder [21].

In animals, fentanyl-induced muscle rigidity induces changes in respiratory mechanics and metabolism that markedly contribute to its toxicity [22]. Rigidity development is potentiated by increases in the dose and the rapidity of its administration. IV fentanyl promoted a significant increase in the electromyographic (EMG) activity recorded from the rat sacrococcygeus dorsi lateralis muscle [23]. Subcutaneous (SC) carfentanil induced dose-related catalepsy in the rat that lasted up to 8 h [24]. The chest and abdominal musculatures were shown to be distressed, resulting in the “wooden chest syndrome”. 

The exact underlying mechanisms supporting chest wall rigidity are unclear. While centrally mediated, this phenomenon does not seem related to the CNS-dependent respiratory drive depression [25]. Various pathways including noradrenergic, glutamatergic, dopaminergic and serotonergic neurons have been implicated. The effects of fentanyl microinjection into the rat locus coeruleus, which increased the EMG activity of the caudal lateral extensor and gastrocnemius muscles considered as a correlate of opioid-induced muscular rigidity, were inhibited with the alpha 1-adrenoceptor blocker prazosin pretreatment, supporting the involvement of a coerulospinal noradrenergic pathway in fentanyl-induced muscular rigidity in rats [26]. The activation of the EMG signals recorded from the sacrococcygeus dorsi lateralis muscle following fentanyl microinjection into the locus coeruleus was also inhibited by the intrathecal administration of various N-methyl-D-aspartate (NMDA) and non-NMDA receptor antagonists [27]. These observations suggested the involvement in fentanyl-induced muscular rigidity of the coerulospinal glutamatergic pathway and both NMDA and non-NMDA receptors in the spinal cord in addition to the ceorulospinal noradrenergic mechanism. The implication of dopaminergic neurons in the basal ganglia, such as the caudate nucleus and nucleus raphe pontis in the reticular formation, was additionally suggested based on the observation of fentanyl-induced alterations in dopamine metabolism in these CNS areas [28]. Mu-opioid receptor antagonization in the locus coeruleus using neurotensin, an amino acid neuropeptide exhibiting significant interactions with the dopaminergic system, resulted in the suppression of fentanyl-induced rigidity in the rat [29]. Although probably not involved physiologically, centrally administered neuropeptide Y attenuated fentanyl-induced rigidity by acting in the same CNS area [30]. A rat mechanography study showed that serotonergic transmission is also involved in fentanyl-induced rigidity, presumably via the 5-HT_1A_ receptors [31]. Finally, a rat study using immunofluorescent staining with a polyclonal antiserum implicated more precisely the G_(o)_ α-subtype of the guanine nucleotide-binding regulatory protein in the underlying signal transduction process [32]. K^+^ channel activation and/or L-type Ca^2+^ channel inhibition secondary to G_o_α protein triggering may additionally contribute to the signal transduction process [33]. 

## 4. Specificities of the Main Fentanyl Analogs

### 4.1. Carfentanil

Preclinical investigations with carfentanil are limited. Mice exposed to 0.4 mg/m^3^ of carfentanil by inhalation during 15 min developed respiratory depression with a marked decrease in V_E_, which was sustained for 24 h after the exposure [34]. Similarly, mice exposure to 6 or 60 mg/min/m^3^ of carfentanil yielded a significant decrease in V_E_ [35]. In another study conducted in African green monkeys, the median effective dose (ED_50_) of SC carfentanil for bradypnea and/or loss of posture was determined at 0.71 μg/kg (95% confidence interval, 0.58–0.87) [36]. This estimate well fitted the experimental data available on carfentanil in other laboratory non-human primate studies. In female rhesus monkeys, respiratory depression was produced at IV doses of 0.6 and 1.0 μg/kg [37]. In Rocky Mountain wapiti (*Cervus elaphus nelsoni*), a regimen combining IM 10 mg/kg carfentanil and 0.1 mg/kg xylazine (administered for anesthesia) decreased the RR, with complete reversion using naloxone administered 10 min later [38]. Carfentanil was found to be the major cause of hypoxemia, as shown by the significant improvement in PaO_2_ after naloxone administration.

### 4.2. Alfentanil

Using microiontophoresis, the alfentanil-induced depressant responses on single rat brain stem respiratory and non-respiratory neurons were shown to be slow, shallow and prolonged, while the responses to the other fentanyl analogs were rapid in onset and short in duration [39]. Using impedance plethysmographic respiratory waveform analysis in the rat, SC 500 µg/kg alfentanil increased the expiratory diaphragm EMG activity while decreasing its inspiratory activity [40]. These modifications in diaphragm function were complemented by a substantial respiratory depression. The effects on EMG activity were greater in the diaphragm than in the intercostal muscles. In rhesus monkeys, alfentanil caused a dose-dependent depression of V_E_ [41]. Quadazocine, a selective mu-opioid receptor antagonist, caused a shift to the right of the dose–effect curves for multiple parameters including RR, V_T_ and V_E_. An arterial blood gas study using multiple alfentanil doses (3, 30, 60 and 120 µg/kg) in canines found a significant short-term decrease in PaO_2_ and an increase in PaCO_2_, most prominently with the 30 and 120 µg/kg doses [42]. In a study designed to examine the cardiorespiratory response to medetomidine in combination with alfentanil in the rat, alfentanil administration decreased the arterial pH resulting ultimately in mixed respiratory plus metabolic acidosis [43]. The acidosis promptly reversed following the injection of 1 mg/kg of naloxone. Interestingly, alfentanil had no significant effects on the respiratory burst studied in vitro in neutrophils, even at higher concentrations than those encountered in in vivo conditions [44]. Of note, multiple other factors can influence the alfentanil-related respiratory effects. For instance, *E. coli* endotoxin was shown to antagonize the alfentanil-induced respiratory depressant effects by promoting its distribution (increase in its volume of distribution) when infused over 10 min [45].

### 4.3. Sufentanil

The specific sufentanil-related effects on ventilation have been poorly investigated. In the dog, sufentanil administration decreased PaO_2_ to 55.0 mmHg, while PaCO_2_ rose to 44.7 mmHg [46]. Similarly, sufentanil administration to rats resulted in an early increase in PaCO_2_ at lower doses and a secondary decrease in PaO_2_ and SaO_2_ at higher doses [47]. The epidural injection, in contrast to the SC injection, of equipotent analgesic doses of morphine, meperidine, fentanyl and sufentanil did not produce significant respiratory effects [48,49,50]. However, when combined with other compounds, the sufentanil-related neuro-respiratory effects were markedly enhanced (Table 1) [47,48,51,52,53]. 

## 5. Reversal of the Neurorespiratory Toxicity Induced by Fentanyl and Analogs

### 5.1. Effects of Naloxone

Opioid overdose rescue is based on the rapid administration of life support and, if available, of naloxone, the antidote of reference. However, due to the rapid onset of fentanyl-induced respiratory depression, attempts to resuscitate patients poisoned with fentanyl or analogs using naloxone may not be effective [54]. These synthetic opioids may require higher and occasionally reiterated naloxone injections to reverse the respiratory depression successfully [55]. Usually, the naloxone dose suitable to reverse opioid-induced CNS depression depends on several factors such as the potency and the dose of the opioid molecule and the chronicity of opioid exposure that may have resulted in tolerance development. Therefore, taking into consideration the high potency of fentanyl and analogs, larger naloxone doses are expected to be required to reverse the CNS depression [56]. A study performed in Rhesus monkeys using positron emission tomography imaging to determine mu-opioid receptor occupancy by naloxone showed that higher doses of IM naloxone led to higher receptor occupancy, useful to improve the reversal of overdoses due to potent opioids such as fentanyl and analogs [57]. 

A lack in CNS depression reversal was highlighted in different animal models receiving fentanyl analogs with usual naloxone doses used to reverse morphine-induced toxicity [35]. In a non-sedated rat model using open-flow plethysmography, severe hypoxia resulting from CNS depression following fentanyl overdose was shown to oppose an unprompted breathing rhythm uniquely opposed by the animals to the effects of a second re-administration of fentanyl [17], which seemed lifesaving but could avert the reversal effects of high-dose naloxone. Additionally, naloxone administration was considered non-optimally effective to reverse the “wooden chest syndrome” due to the lack of effects on cholinergic and noradrenergic sites [58].

Reversal may differ from one fentanyl analog to another. In a conscious rabbit model with arterialized venous blood analysis and RR and V_E_ measurements, naloxone was shown to be more effective in reversing alfentanil than fentanyl effects [59]. In a micro-iontophoresis study examining the effects of fentanyl analogs on rat respiratory and non-respiratory brain stem neurons, naloxone reversed or blocked the slow responses produced by fentanyl and three analogs (sufentanil, lofentanil and alfentanil) but was ineffective on most fast responses. With combined responses, the slow component was blocked, leaving the fast response unaffected [39].

Animal models established the dose-dependent naloxone-related reversal of fentanyl toxicity. In a rabbit study performed in the 1980s, naloxone reversed all residual neurorespiratory effects of fentanyl by antagonizing its related ventilatory, hemodynamic, biological and chemical responses [60]. However, in a more recent mouse study using three IP naloxone doses (0.3, 1 and 3 mg/kg), only the highest dose was effective to reverse fentanyl-induced respiratory depression, in contrast to what observed for morphine [12]. In another mouse model investigating inhaled carfentanil-induced alterations in breathing dynamics, V_E_ was slightly improved using naloxone, with limited dose-dependent effects [34]. The IM 0.05 mg/kg naloxone did not sufficiently increase V_E_ to reach the values of the control animals within 24 h, although clinical manifestations of toxicity and the duration of respiratory depression in the mice were diminished [35]. Even though an increase in V_E_ was observed after naloxone injection, no modifications were identified in the duty cycle, a measure of ventilatory timing [34]. Overall, naloxone alleviated the volumetric aspects of carfentanil-induced respiratory toxicity, while respiratory timing remained unchanged throughout post-exposure observation. All these experimental findings clearly question the optimal dose regimen of naloxone to reverse neurorespiratory toxicity and all its components attributed to fentanyl and analogs, as debated in humans [8,9]. Interestingly, naloxone methiodide, a naloxone formulation unable to cross the BBB in rodents, blocked the fentanyl-induced deleterious effects on the alveolar–arterial gradient and V_T_, preventing deleterious arterial blood gas effects. Recently, a form of naloxone (NX-90) synthetized to reverse a fentanyl-induced overdose when given intranasally, showed an improved pharmacodynamic profile, including greater effects on the restauration of the respiratory and heart rates compared to standard naloxone in rats [61].

### 5.2. Alternative Targeted Strategies

If used to reverse opioid-induced toxicity in patients admitted with respiratory depression attributed to an opioid analgesic overdose, naloxone may also suppress analgesia, a critical issue in these patients suffering from pain. Moreover, if opioid-related respiratory depression is over-antagonized, acute withdrawal syndrome may occur. Therefore, investigating alternative pathways to reverse the opioid-related neurorespiratory toxicity while preserving analgesia is crucial. 

The accentuation of α-amino-3-hydroxy-5-methyl-4-isoxazolepropionic acid (AMPA) receptor-mediated conductance with an ampakine such as CX717 countered the opioid-attributed depressant effects in the pre-Bötzinger complex, allowing protection against fentanyl-induced ventilatory depression and the risk of fatal apnea [62]. Similarly, selective delta-opioid receptor antagonists appeared highly effective to reverse the respiratory depressant effects of potent opioids, while maintaining analgesia [63]. Other opioid receptor antagonists such as methocinnamox showed a greater duration of action than naloxone (up to 2 weeks when administered SC), useful to prevent the re-emergence of fentanyl overdose-related re-narcotization [64]. Another strategy to prevent fentanyl-induced respiratory depression proven effective in mice combined fentanyl with a cannabinoid B2 (CB2) receptor agonist (LY2828360), known to attenuate the development of opioid-induced respiratory depression without recruiting the β-arrestin signaling pathway [65]. 

Addressing muscle rigidity to limit opioid-induced respiratory depression represents another major objective [7]. The α1-adrenoreceptor agonist prazosin was effective to improve rat V_T_ and overall oxygenation [26]. The α_4_β_2_ nicotinic acetylcholine receptor activation was also effective in advancing pain control while limiting respiratory depression in the presence of opioid overdose in the rat [66]. Additionally, and despite unclear mechanisms of action, serotoninergic 5-HT_1A_ receptor agonists decreased opioid-attributed ventilatory depression in different models [67,68,69]. Befiradol, a highly selective 5-HT_1A_ receptor agonist, effectively reversed fentanyl-induced sedation and ventilatory depression; however, simultaneous alteration in analgesia and distorted baseline ventilation, nociception and behavior were reported [70]. Pre-treatment with the protein kinase A (PKA) inhibitor H89 was demonstrated to limit the fentanyl-induced effects on RR, T_I_ and T_E_ [71]. Similarly, pre-treatment with the G protein-gated inwardly rectifying K^+^ (GIRK) channel blocker tertiapin-Q reversed the fentanyl-induced effects on RR and T_I_, thus identifying PKA and GIRK as prospective targets for fentanyl-related respiratory depression therapy. Countering the opioid-induced effects in the Kölliker–Fuse/parabrachial complex (KF/PB), a system that plays a vital role in RR control, chemosensory reflex control, eupneic respiratory pattern formation and hypoglossal motor output, all failing in opioid-induced respiratory depression [72,73,74], was shown to be sufficient to restore the phasic respiratory output at a rate similar to that in pre-fentanyl administration conditions [75]. 

Some of these targeted strategies were also tested using fentanyl analogs. A rat study using loss of righting reflex records, whole-body plethysmography and arterial blood gas measurements investigated the effectiveness of nalmefene versus naloxone in countering carfentanil-induced neurorespiratory depression [76]. Nalmefene more effectively countered carfentanil-induced CNS depression, thus appearing as more appropriate to antagonize carfentanil-related toxicity. Another study speculated about the role of δ-opioid receptors in modulating or counteracting the ventilatory depression produced by mu-opioid receptors, as observed with delta-opioid ligands such as naltrindole, which alleviated alfentanil-related respiratory depression without altering its antinociceptive effects [77]. 

Monoclonal antibodies (mAbs) were also used to reverse synthetic opioid-related neurorespiratory toxicity. A lead mAb (6A4mAb) successfully reversed fentanyl-induced antinociception, similarly to naloxone but with a relatively longer half-life of effect duration [78]. Passive immunization was demonstrated effective in limiting death after high-dose fentanyl administration (above the LD_50_). Rat studies demonstrated that mAbs can counteract toxic fentanyl effects in both pre- and post-exposure scenarios and that naloxone co-administration could provide a greater protection [79]. A pharmacokinetic–pharmacodynamic simulation study extrapolating data to humans was also performed, showing a sharp reduction in ventilation toward apnea followed by a slow restoration of ventilation, with a return to 40% of baseline ventilation after 20 min in a 60 kg person receiving a lethal IV dose of 3 mg of fentanyl [78]. The initial fentanyl-induced drop in ventilation could also rapidly (3 min) return to 50% of baseline ventilation if fentanyl was administered to a person pretreated 24 h before with a 500 mg dose of fentanyl antibodies. Novel murine-derived anti-fentanyl mAbs were selected thereafter based on their high affinity to fentanyl (binding association rate constant of >1 nmoL^−1^·s^−1^ and dissociation constant of <0.7 h^−1^), which could predict their efficacy to block fentanyl-induced effects in mice [80]. Currently, at least two humanized anti-fentanyl mAbs deserve clinical testing to assess their potential to treat fentanyl toxicity. 

Finally, fentanyl vaccines may provide an attractive approach to mitigate fentanyl-attributed adverse effects and toxicity. In the rat, dose–response curves of respiratory effects were shifted to the right with the vaccine, without affecting the ability of naloxone to reverse the respiratory depression [81]. Vaccinated rats showed improved physiological parameters including oxygen saturation and heart rate after exposure to fentanyl compared to unvaccinated rats [82]. In comparison to the controls, decreased post-exposure drug levels in the brain were observed in rats vaccinated with F1-CRM, a supported candidate vaccine against fentanyl and selected analogues. Lead vaccine formulations were highly effective in reducing the respiratory depression caused by fentanyl and other analogs, namely, sufentanil and acetylfentanyl [83], while due to their selectivity, they did not interfere with the pharmacological effects of commonly used anesthetics nor with methadone, naloxone, oxycodone or heroin [84]. Preclinical data obtained in rodents clearly encourage the translation of the fentanyl vaccine strategy to humans to fight against use disorders and toxicity.

## 6. Conclusions

In the shadow of the current opioid epidemic, the medical and recreational use of fentanyl and analogs is rising, with major concerns regarding their neurorespiratory toxicity, the counterpart of their major pharmacological potency, which contributes to the threatening trend of increasing morbidities and mortality from overdose. Understanding the exact specific mechanisms involved in synthetic opioid-induced neurorespiratory effects such as the “wood chest syndrome” is essential to attempt lowering the risks. Based on animal models, various non-opioidergic pathways involved in neurorespiratory toxicity have been identified. Data suggest that higher and repeated naloxone doses are required to reverse the neurorespiratory effects of fentanyl and analogs in comparison to morphine. Predominant naloxone-attributed effects are observed on the slow-response neurons, with a dose-dependent effectiveness to reverse CNS depression. While various pathways could be involved, determining how opioid-insensitive respiratory neurons can quickly escape from fentanyl-induced respiratory depression to generate and maintain the respiratory activity appears as a promising strategy to investigate effective antidotes able to prevent or reverse the neurorespiratory toxicity without altering the analgesic effects of fentanyl and analogs. Research to identify new pathways to target, willing to effectively reverse synthetic opioid-attributed neurorespiratory toxicity, has now become a public health priority due to the continued increase in opioid overdose worldwide.

## Figures and Tables

**Table 1:** Effects of sufentanil drug combinations on respiratory depression.

Combination		Pharmacological Class	Animal Model	Observed Effects
Sufentanil	U-50488H (co-administration)	selective κ-opioid agonist	Rat	Mild increase in PaCO_2_ at the highest tested dose, suggesting that μ- and κ-opioid receptor agonists are more beneficial if combined than if administered alone [46]
Sufentanil	Chlordiazepoxide (pretreatment)	Benzodiazepine	Rat	More profoundly depressed ventilation [47]
Sufentanil	Medetomidine (co-administration)	α2-adrenergic agonist	Rat	Noticeable respiratory depression 30 min post-administration: <50% of resting RR and SaO_2_ reduced to half [50]
Sufentanil	Nimodipine (pretreatment)	Dihydropyridine calcium channel blocker	Rat Cat	Potentiating effects (respiratory rate and tidal volume) [51]
Sufentanil	Nimodipine (co-administration)	Dihydropyridine calcium channel blocker	Rat	No additional potentiating effects but counteraction of tolerance to respiratory depression [51]
Sufentanil	Bay K 8644 (co-administration)	L-type calcium channel agonist	Cat	Partial antagonization [52]

## Data Availability

Data sharing not applicable.
